# An Assessment of Indoor Air Quality before, during and after Unrestricted Use of E-Cigarettes in a Small Room

**DOI:** 10.3390/ijerph120504889

**Published:** 2015-05-06

**Authors:** Grant O’Connell, Stéphane Colard, Xavier Cahours, John D. Pritchard

**Affiliations:** 1 Fontem Ventures B.V., Barbara Strozzilaan 101 12th Floor, HN Amsterdam 1083, The Netherlands; E-Mail: grant.oconnell@fontemventures.com; 2 SEITA–Imperial Tobacco Group, 48 rue Danton, Fleury-les-Aubrais 45404, France; E-Mails: stephane.colard@fr.imptob.com (S.C.); xavier.cahours@fr.imptob.com (X.C.); 3 Imperial Tobacco Limited, 121 Winterstoke Road, Bristol BS3 2LL, UK

**Keywords:** e-cigarette, indoor air quality, bystander exposure, exhaled aerosol, ambient air

## Abstract

Airborne chemicals in the indoor environment arise from a wide variety of sources such as burning fuels and cooking, construction materials and furniture, environmental tobacco smoke as well as outdoor sources. To understand the contribution of exhaled e-cigarette aerosol to the pre-existing chemicals in the ambient air, an indoor air quality study was conducted to measure volatile organic compounds (including nicotine and low molecular weight carbonyls), polycyclic aromatic hydrocarbons, tobacco-specific nitrosamines and trace metal levels in the air before, during and after e-cigarette use in a typical small office meeting room. Measurements were compared with human Health Criteria Values, such as indoor air quality guidelines or workplace exposure limits where established, to provide a context for potential bystander exposures. In this study, the data suggest that any additional chemicals present in indoor air from the exhaled e-cigarette aerosol, are unlikely to present an air quality issue to bystanders at the levels measured when compared to the regulatory standards that are used for workplaces or general indoor air quality.

## 1. Introduction

In recent years, the use of electronic cigarettes (also termed “vaping”) has increased significantly worldwide with such products gaining acceptance with consumers as an alternative to traditional tobacco products. A report published in July 2014 by Action on Smoking and Health estimated as many as 2.1 million adults in the UK currently use electronic cigarettes (e-cigarettes) [1]. E-cigarettes are battery-powered devices that deliver vaporized nicotine, propylene glycol and/or glycerol and flavorings to users from an “e-liquid” [2,3]. They do not contain tobacco or require combustion [2,3]. E-cigarettes are available in many different configurations; the two principal distinctions being “open” systems which can be refilled by the consumer (e.g., tank systems) or “closed” systems (e.g., replaceable cartridges pre-filled by manufacturers) [3]. When the user takes a puff on the product, a heating element is activated converting the e-liquid in the cartridge into an aerosol that the user holds in the mouth or inhales.

With the increasing prevalence of e-cigarettes, there is growing discussion amongst public health organizations and the scientific community as to whether the aerosol exhaled following use of such products has implications for the quality of air breathed by bystanders through so-called “passive vaping”, akin to that reported for environmental tobacco smoke from combusted tobacco products [2,3,4,5,6]. In recent years, there has been conflicting and, at times, confusing information presented to the public regarding the potential risks to bystanders from exhaled e-cigarette aerosol [5,7]. There are calls, including by some government bodies, to prohibit the use of e-cigarettes in workplaces and enclosed public spaces [5,7]. Equally, other organizations and researchers have stated that any regulation on using such products in enclosed public spaces requires an established evidence base, which is limited at this time [2,8].

Airborne chemicals in the ambient air which can impact indoor air quality arise from a wide variety of sources such as those infiltrating from outdoor sources (e.g., vehicle fumes), cooking, burning fuels and tobacco, and (scented) candles [9]. Other sources include emissions from construction materials and furniture, use of air fresheners and cleaning products as well as other consumer goods products like personal care products [9]. To date, there is limited data on the impact of exhaled e-cigarette aerosol on indoor air quality.

Of the few studies that have been undertaken to investigate the impact of e-cigarette emissions on indoor air quality, it has been reported that nicotine, propylene glycol, glycerol (the components of e-liquids), amongst other chemical compounds including volatile organic compounds, low molecular weight carbonyls, polycyclic aromatic hydrocarbons and trace metals, may be released into the air during use of e-cigarettes [10,11,12,13,14,15]. As no validated, standardized protocol is available for measuring exhaled e-cigarette emissions, the limited number of analytical investigations published above differ in environmental conditions and experimental set-up making it difficult to compare their findings and to determine the impact of e-cigarette use on the indoor ambient air. It is also questionable to compare results from smoking machine generated aerosol released into a room [12] with aerosol generated from human subjects [13] due to the changed chemistry and physical properties of the aerosol upon exhalation. Other factors include differences in the type of e-cigarette device used (“closed” *vs*. “open” system), the e-liquid composition, and the e-cigarette consumers’ individual puffing topography, *i.e.*, number of puffs, interval between puffs, puff duration, inhalation volume and depth of inhalation. It has been reported there is wide variations in the quality of e-cigarettes which may also impact measured emission values [16]. Taken as a whole, there is a clear need for studies evaluating indoor air quality before, during and after e-cigarette use to provide important information on the impact of e-cigarettes on indoor air quality and therefore bystander exposures under real-life conditions [17].

In this study, we performed an assessment of indoor air quality before, during and after *ad libitum* use of a disposable ‘closed’ system e-cigarette (Puritane™; manufacturer, Fontem Ventures B.V., Amsterdam, The Netherlands) by human subjects in a naturally ventilated meeting room. Within this study, we analyzed the airborne concentrations of volatile organic compounds (VOCs) including nicotine and low molecular weight carbonyls, polycyclic aromatic hydrocarbons (PAHs), tobacco-specific nitrosamines (TSNAs) and trace metals. To assess indoor air quality and to provide a context for potential bystander exposures, we compared these findings with Human Criteria Values including UK and other general indoor air quality guidelines or workplace exposure limits (WELs), where available. The experimental approach presented here may also be useful to compare the chemicals released into the ambient air from different e-cigarettes used in different indoor environments.

## 2. Experimental Section

### 2.1. Study Design

**Figure 1:**
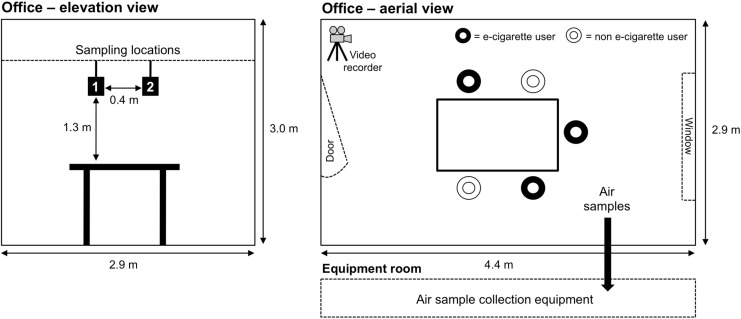
The layout of the meeting room used in this study (not drawn to scale). Sampling locations and positions of the e-cigarette users and non-users during the meeting are highlighted.

**Figure 2:**
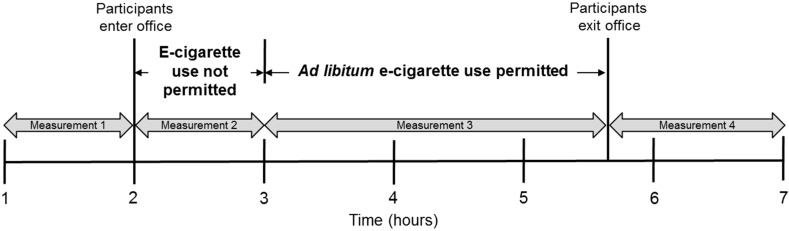
Timeline illustrating when participants entered and exited the office, when e-cigarettes were used and sampling times.

To assess indoor air quality in a real-life environment, a business meeting was conducted in a small meeting room (12.8 m^2^) with five male adult volunteers (three experienced, regular e-cigarette users and two non-users) who had provided written, informed consent. The purpose of this was to create a realistic environment to encourage normal behavior by volunteers, without undue focus on vaping behavior. Smoking or vaping had not occurred in the room previously which was under natural ventilation conditions (*i.e*., no air conditioning and all windows/doors were kept closed during the study). The air exchange rate of the office was confirmed using a standard tracer gas method as described previously by Upton and Kukadia [18]. The internal volume of the room was 38.5 m^3^ and was furnished with a central table and five chairs; a video camera was placed in one corner of the room to record the study and number of puffs taken by the volunteers. Filter assemblies and sampling lines were suspended above the meeting table using metal struts; this served to reduce interference with volunteer behavior. To mitigate potential confounding from operators entering the test space, air samples were drawn using sampling lines into an adjacent room for collection onto tubes or sorbent cartridges specific for the respective chemical parameter being monitored. Samples for metals analysis were taken within the office using filter arrangements. A schematic representation of the room layout, with details of the two independent sampling locations and the positions of the e-cigarette users and non-users is shown in Figure 1. To investigate potential changes in indoor air quality, the ambient air was analyzed before, during and after a 165 min vaping session. Sampling times are shown in Figure 2. During the vaping session, three of the five participants used Puritane™ 16 mg/g disposable Original flavored e-cigarettes (“closed” system; battery capacity, 240 mAh) purchased over-the counter from a number of UK retail outlets. The base e-liquid (1 mL) used in the product consists of mixture of propylene glycol (67% (w/w)) and glycerol (30% (w/w)) in which pharmaceutical grade nicotine (1.6% (w/w); 16 mg/g per product) and small amounts of flavorings are dissolved; a typical e-liquid conformation in the UK. Products were consumed *ad libitum* (*i.e.*, with no restrictions on how to consume the product during the study period) with multiple products available to enable continual vaping during the study period as required; two participants did not use an e-cigarette during the meeting. The study was developed in collaboration with and conducted by an independent, leading UKAS accredited laboratory in the UK with expertise in indoor air quality.

### 2.2. Analysis of Indoor Air Parameters

#### 2.2.1. Indoor Climate

Carbon dioxide was measured continuously using a non-dispersive infrared detector (Q-Trak IAQ monitor, TSI Inc., Shoreview, MN, USA; limit of detection, 9 mg/m^3^). Carbon monoxide was measured continuously using an electro-chemical sensor (Q-Trak IAQ monitor, TSI Inc.; LOD, 1.2 mg/m^3^). Ozone was measured continuously using a UV based photometric analyzer (Ozone Analyzer Model 49C; LOD, 0.002 mg/m^3^ Thermo Environmental Systems, Franklin, MA, USA). Nitric oxide and nitrogen dioxide were measured continuously using a NO_x_ Analyzer (Thermo Environmental Systems Model 42C; LOD, 1.25 mg/m^3^ for nitric oxide and 1.9 mg/m^3^ for nitrogen dioxide). Indoor humidity and temperature were continuously monitored (Q-Trak IAQ monitor, TSI Inc.).

#### 2.2.2. Nicotine

Nicotine was measured in the air by pump sampling maintained at a flow rate of 1 L/min throughout the sampling period through PTFE tubing into XAD2 sorbent tubes (Ref. 226-30-06, SKC Ltd, Dorset, UK). Analysis of exposed tubes was performed by solvent extraction and GC-MS. The LOD for nicotine in air was 7.0 µg/m^3^. Travel blanks were also collected and analyzed.

#### 2.2.3. Volatile Organic Compounds (VOCs)

Sampling and analysis of VOCs was carried out according to the ISO 16000-6 international standard [19]. Pump sampling was maintained at a flow rate of 0.15 L/min throughout the sampling period through PTFE tubing. Travel blanks were also collected and analyzed. The total volatile organic compounds (TVOC) concentration, as used in many indoor air quality guidelines, was calculated as the area of all compounds eluting between, and including, hexane and hexadecane. This is quantified as toluene equivalents, and so the TVOC concentration may be less or more than the sum of the individual VOCs reported. The LODs for each individual VOC were in the range 0.5–1.0 µg/m^3^.

#### 2.2.4. Glycerol

Glycerol was measured in the air by pump sampling maintained at a flow rate of 1 L/min throughout the sampling period through PTFE tubing into XAD7 sorbent tubes (SKC Ltd Ref. 226-57). Analysis of exposed tubes was performed using a thermodesorption unit coupled to by solvent extraction and GC-MS. The LOD for glycerol in air was 150–350 µg/m^3^; this range represents differences in sample durations and therefore sampling volumes. Travel blanks were also collected and analyzed.

#### 2.2.5. Low Molecular Weight Carbonyls

Formaldehyde (methanal), acetaldehyde (ethanal) and acrolein (propenal) were measured in the air by pump sampling maintained at a flow rate of 1.5 L/min throughout the sampling period through PTFE tubing into commercially available purpose-built tubes which contained silica coated with 2,4-dinitrophenyl hydrazine (DNPH). Sampling and analysis of exposed tubes was performed according to ISO 16000-3 international standard [20]. The LOD for carbonyls in air was 2.0 µg/m^3^. Travel blanks were also collected and analyzed.

#### 2.2.6. Polycyclic Aromatic Hydrocarbons (PAHs)

The US Environmental Protection Agency (US EPA) ‘priority list’ of 16 PAHs [21] were measured in the air by pump sampling maintained at a flow rate of 2 L/min throughout the sampling period through PTFE tubing into XAD2 sorbent tubes (SKC Ltd Ref. 226-30-06). Analysis of exposed tubes was performed by solvent extraction and high resolution GC-MS. The LOD for each PAH in air was 1.25 µg/m^3^. Travel blanks were also collected and analyzed.

#### 2.2.7. Trace Metals

The US EPA “Method 29” metals [22], aluminium and phosphorus were measured in the air by pump sampling operating maintained at a flow rate of 6.5 L/min throughout the sampling period into pre-prepared 25 mm filter assemblies (using mixed cellulose ester “MCE” membrane filters). The filters were acid-extracted by digestion in boiling *aqua regia* and the extract analyzed by Inductively Coupled Plasma Optical Emission Spectroscopy (ICP-OES). The LOD for each metal in air ranged from 1.0 to 2.0 µg/m^3^, depending on the metal analyzed. Travel blanks were also collected and analyzed.

#### 2.2.8. Tobacco-Specific Nitrosamines (TSNAs)

TSNAs were measured in the air by pump sampling maintained at a flow rate of 1.5 L/min throughout the sampling period through PTFE tubing into Cambridge filter pads (44 mm diameter) impregnated with potassium bisulphate. Analysis of exposed tubes was performed by solvent extraction and HPLC-MS. The LOD for each TSNA in air was 0.5 µg/m^3^. Travel blanks were also collected and analyzed.

### 2.3. Analysis of Outdoor Air Parameters

Temperature, relative humidity, and levels of ozone and NO_x_ were also monitored outside the building.

## 3. Results and Discussion

Across Europe and North America, consumer interest in electronic vapour (e-vapour) products, including e-cigarettes, continues to grow [1]. While there are some parallels between e-vapour products and conventional tobacco products in terms of product conformation and consumer behaviors, the products themselves are radically different in their design, composition, and the resultant inhaled and exhaled aerosol. As such, product standards and other regulatory measures must take account of this although as a comparatively recent product category, the evidence base on which to establish such regulation is still developing. While e-cigarettes do not combust or generate side-stream emissions, there is currently a debate on whether exhaled e-cigarette aerosols pose a potential exposure risk to bystanders akin to that reported for environmental tobacco smoke from conventional tobacco products [2,3,4,5,6]. In designing the present study, the key aims were to conduct a study under realistic conditions and to examine findings reported previously by other researchers.

### 3.1. Product Use: Puff Rate

From the video footage, the average puff rate across the three e-cigarette users during the 165 min vaping session was calculated to be 3.2 puffs per minute.

### 3.2. Indoor Climate Parameters

The measured room ventilation rate showed a low level of natural ventilation for the size of the office and number of occupants, with an average air exchange rate of 0.8 air changes per hour. The UK Chartered Institute of Building Services Engineers (CIBSE) recommends a ventilation rate of 1.0 air change per hour [23]. However, this level of ventilation is comparable to that previously reported for living rooms in residential properties [24].

The temperature and relative humidity (RH) in the office over the course of the study were in the ranges 22–28 °C and 43%–57% respectively, with both parameters showing a marked increase as a consequence of the room occupation, as would be expected in a small space with limited natural ventilation and no recourse to cooling. The temperature and RH nevertheless remained within the UK Health and Safety Executive (HSE) ranges for acceptable human comfort in an office space [25].

Carbon monoxide was not detected during any of the test periods (vaping or non-vaping). Carbon dioxide (CO_2_) levels increased to a mean level of 5813 mg/m^3^ from a background level of 969 mg/m^3^ during the non-vaping session, with the concentration peaking at nearly 6800 mg/m^3^ during the vaping session. With the windows and door closed, and continuous occupation by five people, this rise in CO_2_ concentrations is to be expected from normal respiration. There were small differences in the concentrations of nitric oxide (NO), nitrogen dioxide (NO_2_) and ozone (O_3_) during the periods of vaping and non-vaping in the meeting room (data not shown). The small variations in the concentrations of these gases were considered to be as a result of the usual changes that occur in the outside atmosphere, which migrate into the building through infiltration.

### 3.3. Volatile Organic Compounds (VOCs; Including Nicotine, Propylene Glycol and Glycerol) and Low Molecular Weight Carbonyls

Table 1 summarizes the results for VOCs, including nicotine, propylene glycol and glycerol (the three principal components of e-cigarette base liquid) and low molecular weight carbonyls. Nicotine is present in most e-liquids and e-cigarettes, and several studies have investigated its presence in the ambient air following product use. After the generation and release of e-cigarette aerosol using a smoking machine into an exposure chamber, McAuley *et al.* [11] reported airborne nicotine concentrations ranging from 0.725 to 8.77 µg/m^3^ following use of rechargeable e-cigarettes with refillable cartomisers containing 24 mg/mL or 26 mg/mL nicotine. Similarly, Czogala *et al.* [12] used three different e-cigarette products containing 16 mg/mL or 18 mg/mL nicotine and found airborne concentrations in an exposure chamber ranging from 0.82 to 6.23 µg/m^3^. Both these studies (and others) used a machine approach to simulate the use of e-cigarettes for estimating potential bystander exposures to exhaled e-cigarette aerosol [11,12,26]. Such an approach does not account for consumer behavior nor the retention of nicotine by the e-cigarette user and so is likely to overestimate airborne nicotine concentrations and potential bystander exposures. In a volunteer study conducted by Schober *et al.* [13], it was found that the nicotine concentration in the ambient air ranged from 0.6 to 4.6 µg/m^3^ during a 2 h vaping session using a rechargeable e-cigarette with refillable tank (“open” system).

**Table 1:** Average indoor air concentrations of VOCs (including nicotine, propylene glycol and glycerol (principle components of the e-liquid)) and low molecular weight carbonyls (µg/m^3^) measured before, during and after use of e-cigarettes from two independent sampling sites.

Chemical Compound	Background (before Participants Enter Room)	Room Occupied (No Vaping)	Room Occupied (Vaping Permitted)	Room Unoccupied (after Participants Leave Room)	Air Quality Guidelines or UK Workplace Exposure Limit as Published (WEL; 8 h Average) (mg/m^3^)	Air Quality Guidelines or UK Workplace Exposure Limit * (WEL; 8 h Average) (µg/m^3^)
	Measurement 1 (µg/m^3^)	Measurement 2 (µg/m^3^)	Measurement 3 (µg/m^3^)	Measurement 4 (µg/m^3^)		
Propylene glycol	<0.5	<0.5	203.6	10.2	UK WEL: 474	474,000
Glycerol	<150	<225	<250	<200	UK WEL: 10	10,000
Nicotine	<7.0	<7.0	<7.0	<7.0	UK WEL: 0.5	500
Isoprene	<0.5	6.2	9.5	<0.5	Not established	Not established
Acetone	1.3	9.2	10.7	1.2	UK WEL: 1210	1,210,000
Propan-2-ol	55.3	13.6	8.0	29.2	UK WEL: 999	999,000
Hexamethylenecyclotri-siloxane	5.3	29.1	13.3	4.4	Not established	Not established
Octamethylcyclotetra-siloxane	<0.5	14.2	3.6	0.9	Not established	Not established
Limonene	2.2	2.1	2.9	1.5	Not established	Not established
Octanal	2.1	3.5	5.4	4.6	Not established	Not established
Decamethylcyclo-pentanesiloxane	6.3	307	460.8	107.5	Not established	Not established
Nonanal	6.3	7.9	10.6	11.0	Not established	Not established
Decanal	2.8	5.7	9.5	11.6	Not established	Not established
2,2,4-Trimethyl-1,3-pentanediol monoisobutyrate	7.7	16.1	17.3	18.0	Not established	Not established
2,2,4-Trimethyl-1,3-pentanediol diisobutyrate	<0.5	<0.5	1.5	2.2	Not established	Not established
Di-isobutyl phthalate	3.5	4.4	2.3	2.8	UK WEL: 5	5000
Formaldehyde	32.0	31.0	37.6	21.0	WHO: 0.1	100
Acetaldehyde	9.0	6.5	12.4	6.0	EU Indoor Air Quality: 0.2	200
Acrolein	<2.0	<2.0	<2.0	<2.0	UK WEL: 0.23	230
Total VOC	65.0	237.0	379.8	129.0	UK Building Regulations: 0.3 (8 h average)	300

* converted to µg/m^3^ to facilitate comparison with analytical findings in this study.

These levels are in general agreement with the theoretical maximum level determined in a recent publication which used a mathematical model to assess the concentration of nicotine in the indoor air following e-cigarette use [27]. However in our volunteer study presented here, there was no measurable increase in nicotine airborne concentrations with vaping when compared with either the no vaping control session or background measurements *i.e*., all measurements were found to be <7.0 µg/m^3^. By way of context, the published UK WEL for nicotine is 500 µg/m^3^ [28]. The low level measured in this study may be attributable to the high retention rate of nicotine in the body, which has previously been reported following inhalation of tobacco smoke [29], as well as some potential loss by deposition [30]. Further research in these areas will be informative.

Propylene glycol and glycerol are principal components of e-liquids and their presence in exhaled e-cigarette aerosol is expected. Concentrations of propylene glycol in the range of 110–215 µg/m^3^ and glycerol in the range of 59–81 µg/m^3^ in the gas phase of emissions have been reported previously [13]. In other studies, McAuley *et al.* [11] observed airborne concentrations of propylene glycol that ranged from 2.25 to 120 µg/m^3^ and Romagna *et al.* [15] reported airborne glycerol concentrations of 72 µg/m^3^.

In our study, during *ad libitum* use of the ‘closed’ system e-cigarettes, propylene glycol in the air of the meeting room increased from <0.5 µg/m^3^ during the no vaping control session to 203.6 µg/m^3^ during vaping. At the end of the vaping session, there was a substantial and rapid decrease in the levels detected (down to 10.2 µg/m^3^). The levels of propylene glycol determined within our study design were below the UK WEL of 474,000 µg/m^3^ set for this chemical [28]. Glycerol, while also expected to be present in the indoor air during the vaping session, could not be detected with satisfactory precision due to the limit of detection (LOD) for this compound (<350 µg/m^3^). Further methodological refinement is required in future work. Nonetheless, it can be established that glycerol in the indoor air did not exceed 350 µg/m^3^ during consumption of the e-cigarettes which is below the UK WEL of 10,000 µg/m^3^ set for this chemical [28].

Total volatile organic compounds (TVOCs) is an analytically based classification for a range of organic chemical compounds present in ambient air or emissions and is used for reporting purposes. In evaluating TVOCs, consideration of the individual compounds is also necessary (Table 1). The background concentration of TVOCs observed in the meeting room ambient air in our study rose from 65 µg/m^3^ to 237 µg/m^3^ upon occupation of the room. While not components of e-liquids, this increase was likely due to the contribution of siloxane compounds arising from the five volunteers. It is well known that siloxanes are widely used in toiletries, deodorants and other personal care products [31]; with increasing room temperature during the study session, release of these and other cosmetic components would likely to have increased. A number of other commonly used aroma compounds (e.g., octanal, nonanal) were also detected at lower levels during the study period. During the vaping phase the TVOC concentrations rose to 379.8 µg/m^3^, conceivably due to further release of siloxanes and exhalation of propylene glycol from the active consumption of the e-cigarettes (see above). Following participant exit from the office, the TVOC concentrations returned to pre-vaping levels. While a WEL has not been established, UK Building Regulations recommend an 8 h average TVOC level of 300 µg/m^3^ [32].

Previous studies have detected the presence of the low molecular weight carbonyls formaldehyde and acetaldehyde in exhaled e-cigarette aerosols [10,13]. It has been reported that potential sources of these compounds in e-cigarette aerosol may arise from the heating or pyrolysis of propylene glycol [33].

Schripp *et al.* [10] evaluated emissions from e-cigarettes after asking a volunteer user to consume three different refillable “open” e-cigarette devices in a closed 8 m^3^ chamber. The authors reported formaldehyde and acetaldehyde in the air of the chamber albeit at significantly lower levels than emissions from a conventional cigarette. Schripp *et al.* [10] concluded that the presence of formaldehyde in the ambient air may be explained by human contamination and not from e-cigarette emissions; it has been previously reported that low amounts of both formaldehyde and acetaldehyde of endogenous origin can be detected in exhaled breath [34]. In addition, it is widely reported that formaldehyde is released from some furniture and fittings, an effect which increases with room temperature and humidity [35]. Taken as a whole, this highlights the importance of appropriate control sampling during air quality studies.

In our study, using a 38.5 m^3^ environment, we observed slight changes in formaldehyde levels from an empty meeting room background value of 32.0 µg/m^3^, to 31.0 µg/m^3^ with occupancy, to 37.6 µg/m^3^ during e-cigarette use. The level fell rapidly to 21.0 µg/m^3^ following vacation of the office by study participants. The WHO has established a guideline indoor air value of 100 µg/m^3^ for formaldehyde [36]. While indicated as a short-term (30 min) guideline to prevent sensitivity or sensitization in both adults and children, WHO has stated that this value is sufficient to prevent long-term health effects, including cancer, since two distinct long term risk assessment models in the review arrived at proposed guideline values of around 210 and 250 µg/m^3^ [36]. The levels of formaldehyde determined within our study design were below WHO Indoor Air Quality guideline value of 100 µg/m^3^ set for this chemical and comparable to range of values typically found in domestic or public spaces [36,37]. Schripp *et al.* [10] and Schober *et al.* [13] both reported formaldehyde levels below the WHO Indoor Air Quality Guideline.

When compared with the non-vaping session, we found acetaldehyde levels changed from a background of 9.0 µg/m^3^ to 6.5 µg/m^3^ after occupation to 12.4 µg/m^3^ during the vaping session. These values and those reported by Schripp *et al.* [10] and Schober *et al.* [13] were well within the EU Indoor Air Quality guideline for acetaldehyde which is set at 200 µg/m^3^ [38].

A further finding in our study was the absence of a measurable increase in acrolein, the pyrolysis product of glycerol [33], in the office air with use of e-cigarettes when compared to control measurements (<2.0 µg/m^3^). This finding is consistent with those findings from Romagna *et al.* [15], who did not detect acrolein in air quality measurements in a 60 m^3^ room during *ad libitum* use of e-cigarettes.

By way of context, it has been reported by the US Environmental Protection Agency (EPA) and others that the burning of candles indoors resulted in a measureable increase of benzene, toluene, formaldehyde, acetaldehyde and acrolein [39]. In air quality measurement studies following their use, formaldehyde levels in the air ranged from 1.0–323.5 µg/m^3^ and acetaldehyde from 1.0 to 74.95 µg/m^3^; reported levels of these two carbonyls measured in our study were substantially less than the maximal values in these studies [9].

For acetone and isoprene, both exhaled breath components [40], there was an increase from baseline during the occupied non-vaping session and active vaping sessions. Isoprene increased from a baseline measurement of <0.5 µg/m^3^ to 6.2 µg/m^3^ during room occupation to 9.5 µg/m^3^ during active vaping. Acetone increased from a baseline measurement of 1.3 µg/m^3^ to 9.2 µg/m^3^ during room occupation to 10.7 µg/m^3^ during active vaping. Following participant exit from the room, the concentrations of both compounds returned to background levels. This indicates that the occupants were the primary source of isoprene and acetone. A UK WEL has not been established for isoprene; acetone levels in all measurements were substantially lower than the UK WEL which is currently 1,210,000 µg/m^3^ [28].

### 3.4. Polycyclic Aromatic Hydrocarbons (PAHs)

Table 2 summarizes the results for the PAHs. Schober *et al.* [13] recently reported airborne concentrations of PAHs increased following e-cigarette use by volunteers, but were still substantially lower than the USA Occupational Safety and Health Administration’s (OSHA) Permissible Exposure Level (PEL) for PAHs in the workplace of 200 µg/m^3^ [41]. In a commentary on this work, Farsalinos and Voudris [42] noted several study limitations including measuring baseline values on different days from the vaping sessions thus changes in airborne PAHs levels may reflect variations in environmental PAH levels and not e-cigarette use. In our study, there was no measurable increase in the airborne concentration of any of the US EPA ‘priority list’ of 16 PAHs during the vaping period (all <1.25 µg/m^3^), which includes seven PAHs classified as probable carcinogens by International Agency for Research on Cancer (IARC) [43,44]. Differences between the current work presented here and the low levels detected by Schober *et al.* [13] may reflect differences in the sensitivity of the methodologies employed, study design and/or differences between products used in the respective studies.

**Table 2:** Average indoor air concentrations of US EPA “priority list” of 16 PAHs (µg/m^3^) measured before, during and after use of e-cigarettes from two independent sampling sites.

Chemical Compound	Background (before Participants Enter Room)	Room Occupied (No Vaping)	Room Occupied (Vaping Permitted)	Room Unoccupied (after Participants Leave Room)
	Measurement 1 (µg/m^3^)	Measurement 2 (µg/m^3^)	Measurement 3 (µg/m^3^)	Measurement 4 (µg/m^3^)
Acenaphthene	<1.25	<1.25	<1.25	<1.25
Acenaphthylene	<1.25	<1.25	<1.25	<1.25
Anthracene	<1.25	<1.25	<1.25	<1.25
Benz[a]anthracene	<1.25	<1.25	<1.25	<1.25
Benzo[b]fluoranthene	<1.25	<1.25	<1.25	<1.25
Benzo[k]fluoranthene	<1.25	<1.25	<1.25	<1.25
Benzo[ghi]perylene	<1.25	<1.25	<1.25	<1.25
Benzo[a]pyrene	<1.25	<1.25	<1.25	<1.25
Chrysene	<1.25	<1.25	<1.25	<1.25
Dibenz[ah]anthracene	<1.25	<1.25	<1.25	<1.25
Fluoranthene	<1.25	<1.25	<1.25	<1.25
Fluorene	<1.25	<1.25	<1.25	<1.25
Indeno[1,2,3-cd]pyrene	<1.25	<1.25	<1.25	<1.25
Naphthalene	<1.25	<1.25	<1.25	<1.25
Phenanthrene	<1.25	<1.25	<1.25	<1.25
Pyrene	<1.25	<1.25	<1.25	<1.25

### 3.5. Trace Metals

Table 3 summarizes the results for trace metals. It has been previously reported in the literature that e-cigarette use may result in the release of metal particles into the ambient air [13,45]. Schober *et al.* [13] reported that levels of aluminium in the ambient air increased 2.4-fold following e-cigarette use. Under the conditions employed in our study, there was no measurable increase in any of the USA “EPA Method 29” metals [22] as well as aluminium and phosphorus during the vaping period compared with the no-vaping control session and background levels. Measurements were all <1.0 µg/m^3^ for antimony, arsenic, barium, cadmium, chromium, cobalt, copper, lead, manganese, mercury, nickel, selenium and zinc; <2.0 µg/m^3^ for aluminium, beryllium, silver and thallium, and <10 µg/m^3^ for phosphorus. Where established for those metals analyzed, all were below UK WELs as shown in Table 4 [28]. Again, the differences in these findings compared to the Schober *et al.* [13] study may be due to differences in the methods employed and/or the design and manufacture processes of the e-cigarette devices used in the respective studies.

**Table 3:** Average indoor air concentrations of US “EPA Method 29” metals (plus aluminium and phosphorous) (µg/m^3^) measured before, during and after use of e-cigarettes from two independent sampling sites.

Chemical Compound	Background (before Participants Enter Room)	Room Occupied (No Vaping)	Room occupied (Vaping Permitted)	Room unoccupied (after Participants Leave Room)	UK Workplace Exposure Limit as Published (WEL; 8 h Average) (mg/m^3^)	UK Workplace Exposure Limit * (WEL; 8 h Average) (µg/m^3^)
	Measurement 1 (µg/m^3^)	Measurement 2 (µg/m^3^)	Measurement 3 (µg/m^3^)	Measurement 4 (µg/m^3^)		
Aluminium	<2.0	<2.0	<2.0	<2.0	10	10,000
Antimony	<1.0	<1.0	<1.0	<1.0	0.5	500
Arsenic	<1.0	<1.0	<1.0	<1.0	0.1	100
Barium	<1.0	<1.0	<1.0	<1.0	0.5	500
Beryllium	<2.0	<2.0	<2.0	<2.0	0.002	2.0
Cadmium	<1.0	<1.0	<1.0	<1.0	0.025	25
Chromium	<1.0	<1.0	<1.0	<1.0	0.5	500
Cobalt	<1.0	<1.0	<1.0	<1.0	0.1	100
Copper	<1.0	<1.0	<1.0	<1.0	1	1000
Lead	<1.0	<1.0	<1.0	<1.0	Not established	Not established
Manganese	<1.0	<1.0	<1.0	<1.0	0.5	500
Mercury	<1.0	<1.0	<1.0	<1.0	0.02	20
Nickel	<1.0	<1.0	<1.0	<1.0	0.1	100
Phosphorus	<10.0	<10.0	<10.0	<10.0	Not established	Not established
Selenium	<1.0	<1.0	<1.0	<1.0	0.1	100
Silver	<2.0	<2.0	<2.0	<2.0	0.1	100
Thallium	<2.0	<2.0	<2.0	<2.0	0.1	100
Zinc	<1.0	<1.0	<1.0	<1.0	Not established	Not established

* converted to µg/m^3^ to facilitate comparison with analytical findings in this study.

### 3.6. Tobacco-Specific Nitrosamines (TSNAs)

Table 4 summarizes the results for TSNAs. Previous studies have reported the presence of TSNAs in the e-liquid or mainstream e-cigarette aerosols [46]. In our study, we sampled the ambient air for the presence of *N*’-nitrosonornicotine (NNN), 4-(methylnitrosamino)-1-(3-pyridyl)-1-butanone (NNK), *N*’-nitrosoanatabine (NAT) and *N*’-nitrosoanabasine (NAB). There was no measurable increase in the airborne concentrations of the four TSNAs analysed during active consumption of e-cigarettes when compared to control measurements (all < 0.5 µg/m^3^).

**Table 4:** Average indoor air concentrations of TSNAs (µg/m^3^) measured before, during and after use of e-cigarettes from two independent sampling sites.

Chemical Compound	Background (before Participants Enter Room)	Room Occupied (No Vaping)	Room Occupied (Vaping Permitted)	Room Unoccupied (after Participants Leave Room)
	Measurement 1 (µg/m^3^)	Measurement 2 (µg/m^3^)	Measurement 3 (µg/m^3^)	Measurement 4 (µg/m^3^)
*N’*-Nitrosonornicotine (NNN)	<0.5	<0.5	<0.5	<0.5
4-(Methylnitrosamino)-1-(3-pyridyl)-1-butanone (NNK)	<0.5	<0.5	<0.5	<0.5
*N’*-Nitrosoanatabine (NAT)	<0.5	<0.5	<0.5	<0.5
*N’*-Nitrosoanabasine (NAB)	<0.5	<0.5	<0.5	<0.5

### 3.7. Study Limitations and Strengths

The key aim of our study design was to replicate a real-life scenario with unrestricted use of a disposable “closed” system product by the vaping volunteers. In doing so, overhead sampling of the ambient air was chosen rather than personal dosimetry approaches to reduce potential confounding of vaping behaviors from intrusive sampling.

Our use of volunteers in conditions designed to replicate those in a real-world situation limited the sample duration and therefore the sensitivity of the some of the methods employed, which were not as sensitive as in some other studies which used a machine generated aerosol. Arguably, if the presence of certain chemicals can only be detected by employment of artificial or atypical conditions, it is reasonable to question the appropriateness of such data. The use of consumers within the study removed many of the issues associated with the use of smoking machine generated aerosols, for example questions around the potential retention of chemicals in the body or that of different machine protocols not replicating product consumption profiles. With regards to the method to measure glycerol in our study, sensitivity was not as low as anticipated. While there could be some scope for reducing the LODs for these and other chemicals further by increasing sampling duration, this would be difficult without introducing other potential confounding factors such as opening and closing meeting doors for refreshment breaks. By excluding opening and closing doors in this study, and bylimiting the air exchange to natural room ventilations, the levels reported in our study are likely to represent an overestimate of normal conditions. The measurement of air exchange and other environmental parameter measurements in the methodology are supportive of this.

Another limitation in this study was the use of a single product; as noted above, other research groups have reported findings that were not replicated in this present study. Such studies used different products which may reflect variations in e-liquid or device quality, sufficient details of which are often not reported. Additionally, given the focus on ambient air, the primary emissions of the analyzed product were not determined in this study, which may be of interest in future work focusing on consumer rather than bystander exposures. Further air quality studies could also investigate other product types as well as different settings and volunteer groups.

The potential issue of cross contamination with cigarette smoke has been noted previously [2]. Given the sensitivity of the methods employed in this study, potential confounding from recent tobacco smoking was minimized. A strength of this study was that the rooms used here had never been smoked in nor were they used for any prior tobacco research.

## 4. Conclusions

The present study offers an indoor air quality assessment by an independent, UKAS accredited laboratory following use of a disposable ‘closed’ system e-cigarette in a real life setting. Since this was not a long-term repeated exposure study; in providing a context, findings were related to indoor air quality guidelines, where available. Our data indicate that exposure of bystanders to the chemicals in the exhaled e-cigarette aerosol, at the levels measured within our study, are below current regulatory standards that are used for workplaces or general indoor air quality. This finding supports the conclusions of other researchers that have stated there is no apparent risk to bystanders from exhaled e-cigarette aerosols [6,11,47].

There has been conflicting and at times confusing information reported regarding the potential risks of bystanders and non-e-cigarette users to exhaled e-cigarette aerosol. The regulatory outlook from a public health perspective currently remains undetermined; there is a clear need for further research in this area to support the development of appropriate product standards and other science-based regulatory measures.
